# Thermo-Mechanical Deformation, Jamming Risk and Life Management of Main Steam Valves in Ultra-Supercritical Steam Turbines: A Short Review

**DOI:** 10.3390/ma19163370

**Published:** 2026-08-07

**Authors:** Weiwei Huang, Guozheng Quan, Hao Shi, Yabing Duan, Yu Wang, Yawei Li, Lin Yang, Quanqiu Jiang, Chunyu Mou, Daojun Zhang, Feng Ding, Haitao Wang

**Affiliations:** 1 National Energy Group Jiaozuo Power Plant Co., Ltd., Jiaozuo 454000, China; 2 Chongqing Key Laboratory of Advanced Mold Intelligent Manufacturing, School of Materials Science and Engineering, Chongqing University, Chongqing 400044, China; 3 Dongfang Turbine Co., Ltd., Dongfang Electric Corporation, No. 666 Jinshajiang West Road, High-Tech Industrial Park, Jingyang District, Deyang 618000, China

**Keywords:** ultra-supercritical steam turbine, main steam valve, 9–12% Cr steel, thermal deformation, creep–fatigue interaction, functional clearance, jamming, finite element analysis, digital twin

## Abstract

Ultra-supercritical (USC) steam turbines combine severe steam conditions with increasingly frequent start-up, shutdown, and load-following operations. Their main steam valves must preserve pressure boundary integrity, sealing, and rapid actuation while non-uniform heating, creep, cyclic plasticity, oxidation, wear, and contact redistribution alter component geometry. However, the relevant evidence remains fragmented across alloy development, component thermo-mechanics, valve aerodynamics, and lifetime monitoring. This short, mechanism-oriented review integrates these domains through a material structure–function framework in which deformation relative to assembly clearance governs jamming risk. It synthesizes evidence on heat-resistant body and surface materials, 9–12% Cr steel stability, weldability and repair sensitivity, and cold, warm, and hot start-up histories. It also evaluates creep–fatigue interaction, contact, flow-induced vibration, multi-physics modeling, validation, uncertainty, monitoring, and digital twins. The synthesis shows that neither peak equivalent stress nor steady-state temperature alone can establish functional reliability. Credible assessment requires temperature-dependent material data, realistic steam-side heat transfer, cyclic constitutive behavior, initial and residual clearances, manufacturing and assembly tolerances, state-dependent friction, uncertainty analysis, and corroborating plant or inspection evidence. The most consequential research needs are valve-level validation datasets, thermal contact testing, function-oriented life criteria, and uncertainty-aware digital twins that jointly inform materials, geometry, and transient operation.

## 1. Introduction

Decarbonization does not immediately eliminate the need for dispatchable thermal generation; it changes the duty imposed on the remaining fleet. Steam cycle power plants increasingly accommodate renewable variability through load following, peak shaving, and rapid restart. These services diversify and increase thermal transients in steam turbine hot components, even when annual operating hours decline [1,2]. Ultra-supercritical (USC) technology improves cycle efficiency through higher steam temperature and pressure. The same conditions intensify creep, oxidation, and transient thermo-mechanical loading, motivating sustained alloy development programs [3].

Main steam valves, including stop, governing, and combined stop-control valves, are critical in this operating transition. They must meter flow stably, maintain high-pressure sealing, and close rapidly on demand. Unlike a nominally uniform pressure vessel, a valve combines thick cast or forged sections with ports, seats, stems, sleeves, bushings, hard facing, bolted joints, and small moving clearances. Steam impingement and partial opening create non-uniform thermal and flow fields. Guide interfaces can then convert small local displacements into large increases in friction or actuation force. Functional failure, such as delayed closure or stem jamming, may therefore precede a conventional rupture or crack growth limit.

**Figure 1:**
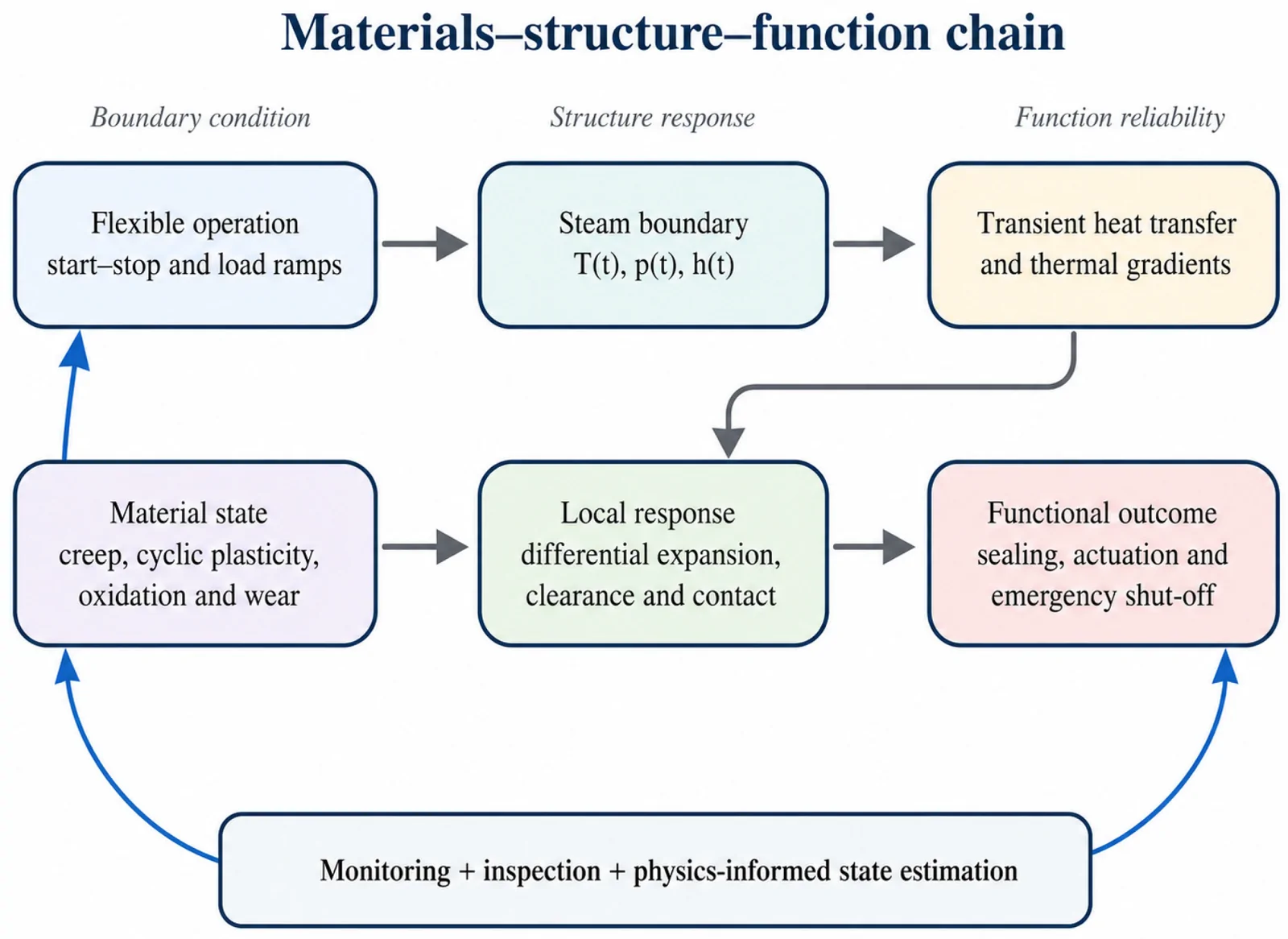
Material structure–function chain for ultra-supercritical (USC) main steam valves. Flexible operation changes the steam boundary and generates transient thermal gradients. Material state and assembly constraints convert those gradients into local deformation, contact, and friction, while monitoring and inspection close the management loop. Original schematic.

Relevant evidence is extensive but compartmentalized. Alloy studies emphasize creep strength and microstructural stability, whereas turbine-transient studies usually address rotors, casings, or nozzle boxes. Valve flow research focuses on capacity, pressure fluctuation, and vibration, while plant life systems generally track component-level stress or fatigue consumption. These studies do not directly resolve the valve-specific question: How do material degradation and transient deformation consume functional clearance, redistribute contact, and impair actuation? This review addresses that gap by treating the main steam valve as a coupled material structure–function system (Figure 1).

Accordingly, the review pursues four linked objectives. First, it identifies the material properties and degradation mechanisms that control valve function. Second, it connects cold, warm, and hot start-up histories to deformation, residual clearance, and jamming. Third, it evaluates the hierarchy of thermal, structural, contact, and reduced-order models. Finally, it defines priorities for material-informed design and digital life management. The organizing principle is a critical synthesis across mechanisms and decision scales, rather than an encyclopedic catalog of individual studies.

## 2. Review Scope and Evidence-Synthesis Method

This article is a narrative, evidence-oriented review rather than a systematic review or meta-analysis. Literature published or available up to 23 June 2026 was identified through Crossref, OpenAlex, and PubMed, where applicable, as well as publisher websites and backward and forward citation tracking. Search concepts combined the terms steam turbine or main steam valve with ultra-supercritical, heat-resistant steel, 9–12% Cr, thermal stress, creep–fatigue, contact, clearance, jamming, control-valve flow, vibration, monitoring, and digital twin. Priority was given to peer-reviewed primary studies, authoritative reviews, current standards, and sources whose methods and figures could be inspected. The literature was synthesized by its causal role in the material structure–function chain, rather than by publication chronology.

The scope covers main stop valves, control or governing valves, combined valves, and closely related reheat valves in supercritical and USC systems. The primary outcome is functional reliability, defined here as retained sealing, guidance, stable actuation, and emergency closure. Evidence was grouped as direct valve evidence, transferable evidence from other thick-walled turbine components, or foundational materials and modeling evidence. Conclusions about jamming were weighed most strongly toward direct valve studies, and transferability is stated whenever inference relies on adjacent components. Purely aerodynamic optimization is considered only when it alters heat transfer, unsteady loading, vibration, or deformation. No unpublished plant data or original life predictions are reported.

Generative artificial intelligence was used to assist with English-language editing, reference-metadata organization, and the production of original schematic figures. The authors independently verified all scientific claims, numerical values, citations, and figure attributions against the cited sources. No primary data were generated, selected, or altered using artificial intelligence.

All tables in this review are original synthesis tables compiled by the authors from the cited literature and through engineering reasoning; they do not reproduce third-party tables. All newly drawn schematic figures are original; when literature-derived numerical values are used, the figure caption explicitly states that the values were replotted or synthesized from the cited source. This statement was added to clarify table provenance, figure attribution, and copyright status.

## 3. Service Conditions and Functional Reliability

### 3.1. Valve Functions, Constraints, and Critical Clearances

A steam valve must satisfy competing requirements. Tight guidance and accurate alignment limit leakage, impact, and vibration, but small clearances leave little tolerance for differential expansion, distortion, oxide growth, or debris. Larger clearances improve resistance to jamming but can increase eccentricity, impact wear, and flow instability. The design objective is therefore not to eliminate deformation. It is to coordinate deformation so that the minimum hot clearance remains positive and the actuation margin remains adequate throughout the service history.

In practice, functional clearance can be quantified as a time-dependent minimum hot-clearance margin, cmin(t), evaluated at each guide, sleeve, seat, and fitted interface. A conservative engineering expression is cmin(t) = c0 + Δctol − Δuthermal(t) − δres(t) − δox(t) − δwear(t) − δdebris(t), where c0 is the measured assembly clearance, Δctol accounts for manufacturing and assembly tolerance, Δuthermal is the relative transient displacement of the mating parts, and δres, δox, δwear, and δdebris represent residual distortion, oxide-scale growth, wear, and debris accumulation. Acceptable operation requires cmin(t) to remain above a plant-specific safe allowance while the actuator force, closing time, leakage rate, and stem position response remain within specified limits.

Therefore, an engineering acceptance criterion should combine at least three measurable quantities: (i) minimum hot-clearance margin or its calibrated proxy, (ii) actuator force or hydraulic pressure margin during opening and emergency closure, and (iii) closing-time repeatability after cold, warm, and hot transients. A valve may satisfy pressure boundary criteria but still be functionally unacceptable if any of these quantities trend toward contact locking.

Several spatial and temporal scales interact. The thick valve body responds over minutes to hours, whereas exposed stems, plugs, and guides can respond much faster. Ports, ribs, flanges, seats, and insulation generate three-dimensional thermal asymmetry. Contact or interference at a sleeve, bushing, seat, or limit ring adds local constraint. Consequently, modest global body displacement can coexist with a critical inward movement that closes a guide clearance.

### 3.2. Cold, Warm, and Hot Start-Up Histories

Start-up categories are usually defined by initial metal temperature or shutdown duration, but their mechanical significance lies in the complete steam–metal temperature history. Cold starts generally create the largest thermal gradients and strain ranges. Warm starts can reduce mismatch and promote coordinated expansion when the trajectory is controlled. Hot restarts begin with retained heat and possible residual deformation. If admitted steam is cooler than the local metal, heat flow reverses and creates a distinct shock pathway. Thus, the smallest incremental temperature rise does not necessarily provide the largest functional margin.

**Figure 2:**
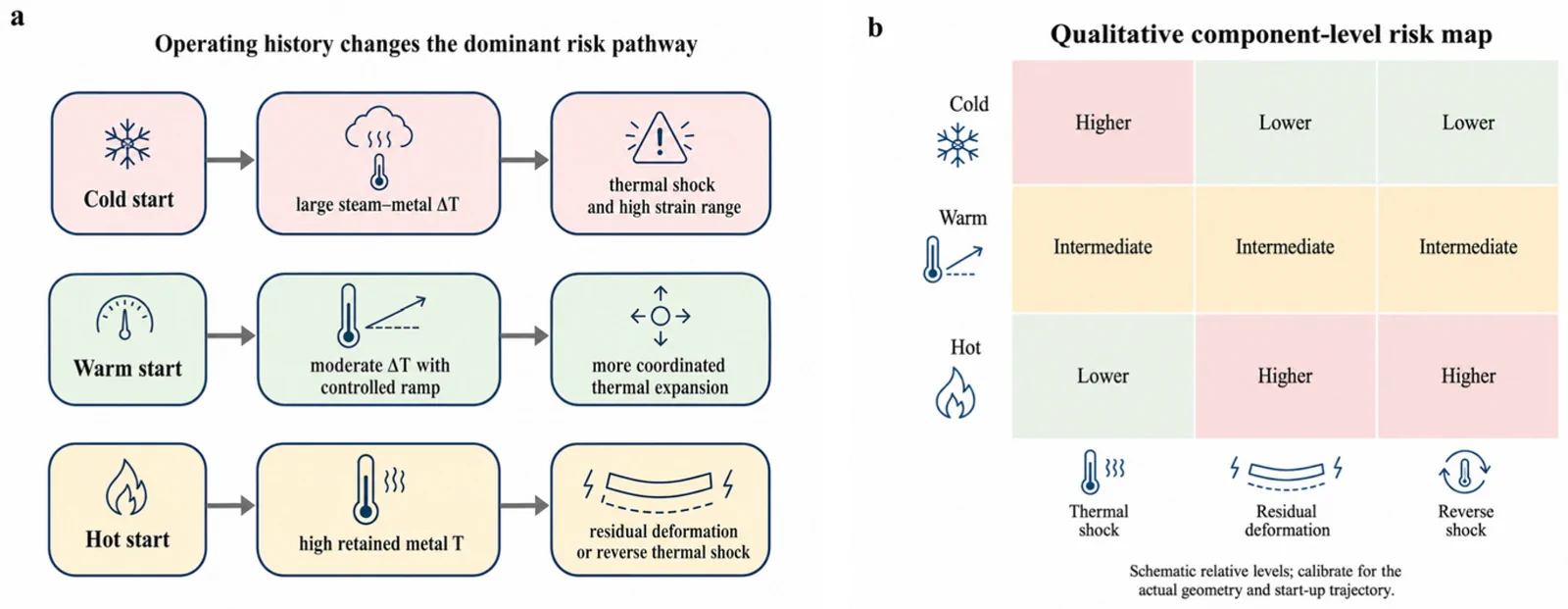
Start-up mode and dominant reliability pathway. (**a**) Cold, warm, and hot histories create different balances among thermal shock, coordinated expansion, and retained deformation. (**b**) Qualitative risk map. The levels are schematic and require component-specific calibration; they are not operating limits.

Figure 2 separates immediate thermal shock severity from residual deformation and reverse shock sensitivity. The resulting ranking is conditional, not universal, because it depends on insulation, shutdown duration, valve opening, steam–temperature matching, material properties, and assembly stiffness. A plant-wide label such as a warm start should therefore not replace a valve-specific estimate of metal temperature, deformation state, and remaining clearance (Table 1).

### 3.3. Structural Life Versus Functional Life

Structural life concerns crack initiation, creep rupture, pressure boundary integrity, and leak-before-break behavior. Functional life concerns whether the valve continues to seal, move, and close within specified time and force limits. These outcomes are coupled but not equivalent. Creep strain that is minor relative to rupture life can still consume a guide clearance measured in fractions of a millimeter. Conversely, a local fatigue crack can threaten integrity without immediately causing jamming. Valve assessment should therefore report damage measures together with displacement, clearance, contact, and actuation variables. This dual criterion provides the evaluative lens for the materials, modeling, and monitoring evidence reviewed below.

The practical relationship between structural and functional life is asymmetric. Structural integrity may remain acceptable when creep strain, oxidation growth, or repair-induced distortion has already reduced the guide clearance below a reliable actuation threshold. Conversely, a small crack indication may require structural assessment even if the valve still moves freely. The review therefore treats functional life as a parallel limit state governed by clearance, contact pressure, friction, and actuator margin, rather than as a secondary consequence of rupture or fatigue damage.

## 4. High-Temperature Materials as a Component System

### 4.1. Body Steels and Microstructural Stability

Valve bodies and chests require a balance of creep strength, toughness, thermal fatigue resistance, oxidation resistance, weldability, castability or forgeability, and inspectability. Low-alloy Cr–Mo–V steels have extensive service experience. Higher steam temperatures have promoted 9–12% Cr tempered martensitic steels and, under more severe conditions, austenitic steels or nickel-based alloys [4,5,6,7]. Alloy selection remains component-specific because thermal conductivity and expansion can influence transient stress as strongly as high-temperature strength.

The creep resistance of 9–12% Cr steels derives from a hierarchical tempered martensitic lath structure. Boundary M23C6 carbides, intralath MX carbonitrides, solute effects, and the dislocation substructure stabilize this architecture. Long-term exposure nevertheless drives recovery, lath widening, and precipitate coarsening or redistribution. Z-phase formation can consume fine MX particles. Laves-phase evolution may initially strengthen the alloy but later promote boundary coarsening or local depletion [8,9,10,11,12,13,14]. Creep degradation is therefore a coupled loss of boundary pinning, dislocation density, and subgrain stability, not a single-phase reaction.

**Figure 3:**
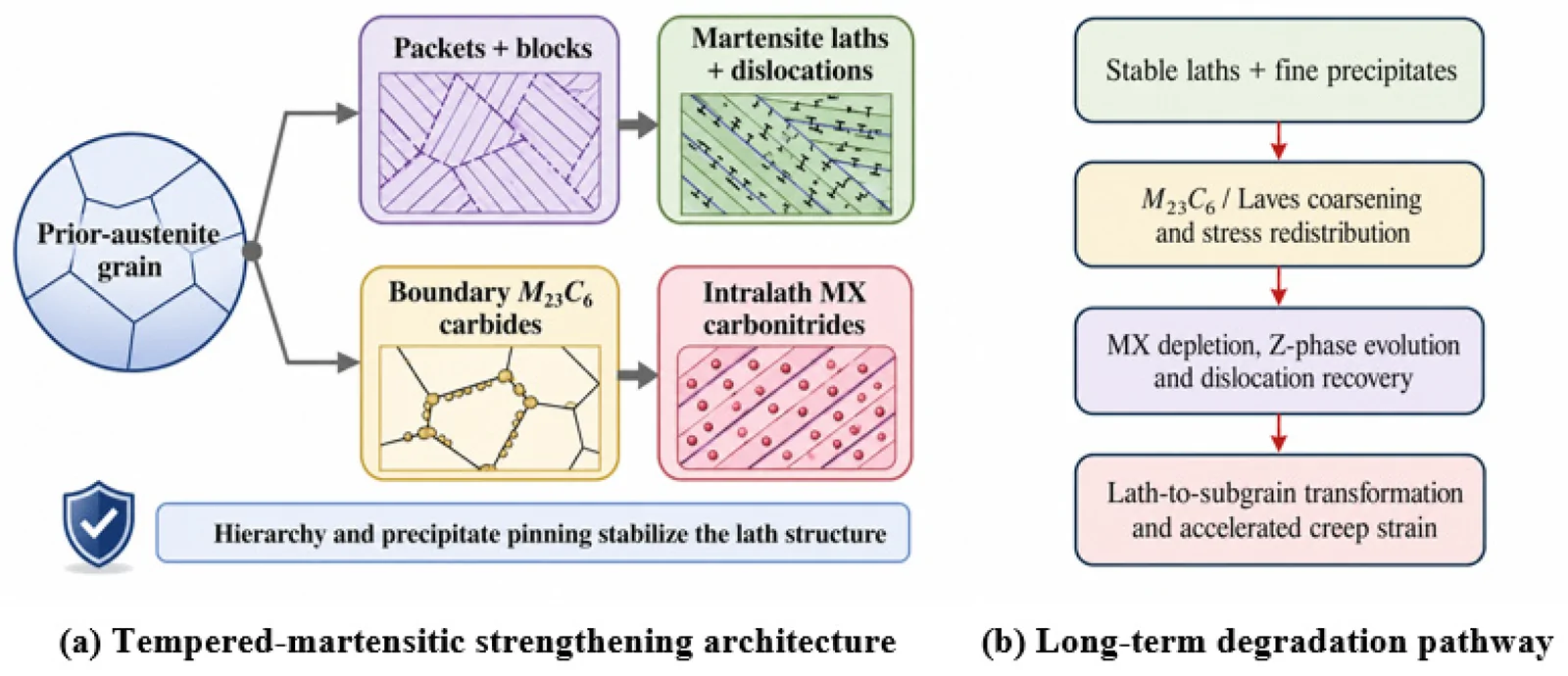
Microstructural stabilization and long-term creep context for 9–12% Cr tempered martensitic steels. Original schematic synthesized from Refs. [7,8,9,10,11,12,13,14]. This figure was generated using ChatGPT (OpenAI; https://chatgpt.com/; accessed on 20 July 2026).

Boron-enriched, low-nitrogen variants, including MARBN-, G115-, and SAVE12AD-type steels, aim to stabilize M23C6 particles and delay recovery. At 650 °C, Dudova reported 100,000 h creep rupture strengths of approximately 80–110 MPa for advanced 9% Cr steels [7]. The corresponding range for P92 and Co-modified P92 variants was about 60–70 MPa. These comparative ranges are not code allowables. They also do not establish valve suitability without evidence on toughness, fabrication, weldments, cyclic response, and dimensional stability (Figure 3).

Rupture strength is therefore an incomplete material-selection metric. Valve function also depends on creep strain accumulation, stress relaxation, and permanent distortion. A material with adequate rupture life can still lose functional margin when a constrained guide or seat accumulates local strain. Property databases should include full creep curves, cyclic stress–strain response, relaxation behavior, and temperature-dependent thermal properties across the transient range, rather than allowable stress alone.

### 4.2. Cyclic Plasticity and Creep–Fatigue Response

Repeated start-stop operation imposes strain-controlled cycles superposed on high-temperature dwell. Tempered martensitic steels commonly soften cyclically as dislocations rearrange, lath boundaries evolve, and recovery proceeds. Constitutive descriptions range from phenomenological cyclic plasticity to microstructure-informed models [15,16,17]. During a hold, stress relaxation and creep alter the internal state encountered on reversal. Linear addition of independently calculated creep and fatigue fractions can therefore be either non-conservative or overly conservative, depending on the load sequence and dwell.

Studies of high-temperature turbine steels show that notch constraint, mean stress, relaxation, and crack closure modify the apparent creep–fatigue interaction [18,19]. Valves add a functional complication: cyclic inelastic strain can change geometry and contact before a crack criterion is reached. Calibration should therefore combine hysteresis and damage data with residual strain measurements obtained under representative ramps and dwell times.

### 4.3. Stems, Guides, Seats, Joints, and Surface Systems

**Figure 4:**
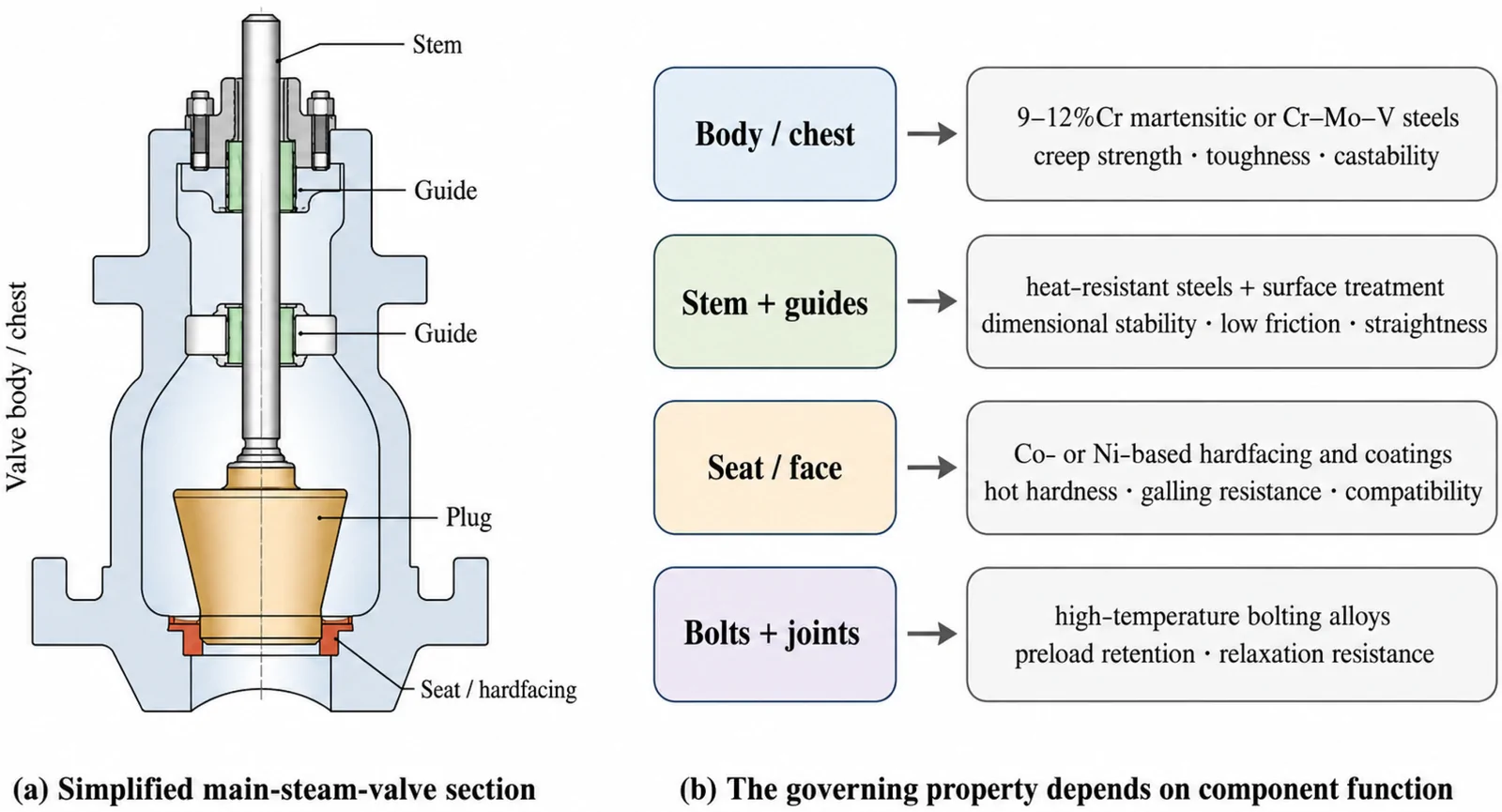
Component-specific materials requirements in a simplified main steam valve.

Stems and guides prioritize straightness, dimensional stability, low friction, and resistance to adhesive and abrasive wear. Seats and sealing faces require hot hardness, erosion and galling resistance, and compatibility with the substrate and counterface. Bolting and fitted joints require preload retention and resistance to relaxation. These functions often favor materials or surface treatments different from those used for the pressure boundary. Figure 4 and Table 2 summarize this component-level materials map and the corresponding materials–function relationships.

Surface selection cannot be reduced to hardness. Thermal expansion mismatch, deposition dilution, residual stress, coating thickness, oxidation, roughness evolution, and debris generation all influence contact. Tribological evidence further shows that friction is a system response, not an immutable material constant [20]. Candidate material couples should therefore be tested in steam at representative temperatures, contact pressures, and sliding amplitudes.

Steam oxidation changes both the load-bearing section and functional clearance. Adherent, slow-growing scales can be protective, but thermal cycling can crack or spall them. Water vapor also alters transport and scale stability relative to dry oxidation [21,22]. At a guide interface, detached scale becomes both abrasive debris and a potential geometric obstruction. Oxidation and tribology must therefore be represented in the same jamming pathway.

Welds and repairs add further heterogeneity. Type IV cracking in the fine-grained or intercritical heat-affected zone of creep strength-enhanced ferritic steels remains a major life assessment concern [23,24]. Even before cracking, weld residual stress and local property gradients can bias distortion and contact. Evidence from ferritic-martensitic alloys developed for reactors and other high-temperature systems is informative, but transfer to valves must account for different environments, fabrication routes, and load paths [25]. Together, these material-state variables define the constitutive, dimensional, and frictional inputs required by a valve-level reliability model.

Weldability should be evaluated at three coupled levels. First, the weld thermal cycle determines HAZ width, intercritical softening, precipitate dissolution, residual stress, and post-weld heat treatment response. Second, the weld metal, HAZ, and base metal form a property gradient system whose weakest zone may govern creep deformation, Type IV cracking, and local distortion. Third, valve function imposes an additional dimensional requirement: weld shrinkage, repair buildup, grinding, hard-facing dilution, and coating thickness must not consume the designed hot clearance or create eccentric contact. Accordingly, procedure qualification for USC valve repairs should include hardness mapping across the HAZ, creep or cross-weld creep evidence where applicable, residual stress or distortion control, metallographic verification after PWHT, and dimensional inspection of the repaired guide or seat interface [23,24,26,27,28,29].

## 5. From Thermal Transients to Functional Jamming

### 5.1. Heat Transfer and Differential Expansion

Transient deformation originates in the steam-side thermal boundary. Local heat flux depends on steam temperature, pressure, velocity, turbulence, valve lift, impingement, and surface condition. Thin or directly exposed regions can heat rapidly, whereas massive external and shielded regions lag. During shutdown or hot restart, the gradient can reverse. A uniform heat transfer coefficient may reproduce the average metal temperature while missing the local gradient that drives a critical inward displacement.

Thermal expansion coefficient, conductivity, specific heat, and elastic modulus vary with temperature and should be represented consistently. Pressure may change quasi-steadily relative to temperature, but contact status changes abruptly when a clearance closes. The decisive output is therefore not maximum von Mises stress alone. It is the time-dependent relative radial and axial displacement between mating components, evaluated against the available clearance.

### 5.2. Creep-Fatigue Accumulation and Residual Deformation

At high temperature, sustained stress produces creep strain while relaxing thermally induced stress. Repeated transients add plastic strain and microstructural recovery, which modify later cycles. A critical region may experience compression during heating, tension during cooling, and a high-temperature dwell under pressure. Finite element studies of high-pressure steam components exposed to thermal shock illustrate why local gradients and cyclic material response must be resolved [30].

Residual deformation is the state variable that links successive cycles. A hot restart begins from the temperature, stress, contact, and geometry left by the previous shutdown, not from the original assembly state. Single-cycle analysis can underpredict clearance loss when creep or ratcheting accumulates and overpredict it when shakedown occurs. Multi-cycle simulation should continue until residual displacement stabilizes, develops a clear trend, or can be updated by a validated reduced-order model.

### 5.3. Contact, Friction, Oxidation Debris, and Functional Clearance

**Figure 5:**
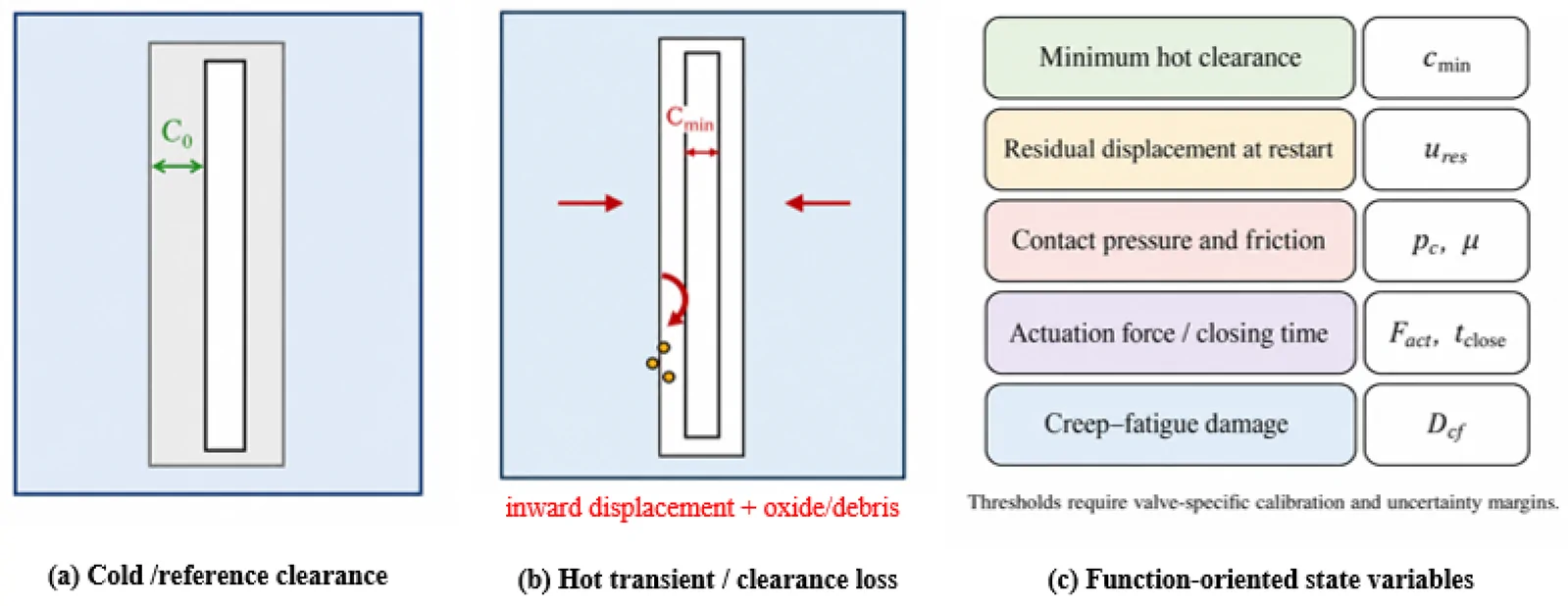
Functional-clearance framework for valve jamming. Green: cold/reference clearance; red arrows: inward displacement and clearance loss; yellow dots: oxide/debris; colored boxes: function-oriented state variables.

Jamming is a localized, contact-driven functional failure. It may result from direct thermal closure of radial clearance, residual distortion at restart, pressure-dependent friction, misalignment, galling, or oxide and wear debris. A small contact patch at an upper guide or limit ring can dominate actuator demand even when average body stress remains moderate. Figure 5 therefore represents jamming through function-oriented state variables rather than a single stress contour.

The interaction among creep, cyclic plasticity, oxidation, and contact is path-dependent. Creep and ratcheting generate residual displacement; oxidation and wear change the effective geometry and surface roughness; contact pressure amplifies friction and local heat transfer; and increased friction feeds back into actuator force and guide wear. These mechanisms should therefore be propagated as coupled state variables, not as independent safety factors added only at the end of a calculation (Table 3).

### 5.4. Flow-Induced Vibration as an Interacting Risk

Control-valve flow depends strongly on lift, pressure ratio, plug and seat geometry, and upstream disturbances. Full-scale tests, model experiments, and computational fluid dynamics (CFD) have documented nonlinear flow characteristics, separation, and pressure fluctuations in steam turbine valves [31,32,33,34]. Strainers, spindle shape, and chamber geometry can reorganize jets and coherent structures [35,36,37,38,39].

Flow-induced vibration and thermal jamming are distinct mechanisms, but they can reinforce one another. Vibration and impact accelerate wear, damage guides, and change effective clearance. Thermal distortion, in turn, alters eccentricity and the local flow field. Reviews identify jet impingement, asymmetric loading, vortex shedding, separated flow, and acoustic or cavity resonance as recurrent excitation classes [40,41]. Flexible service assessment should therefore couple these phenomena whenever the predicted deformation materially changes the flow or contact state.

**Figure 6:**
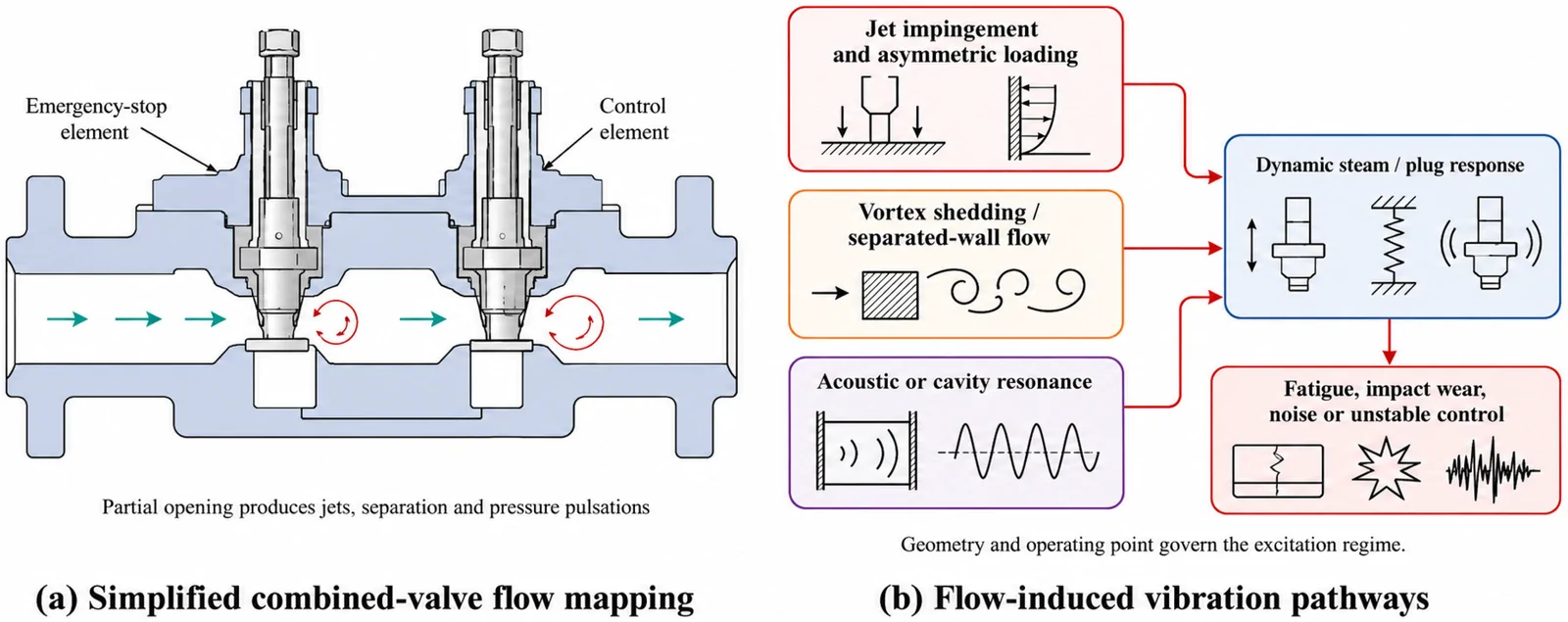
Flow mapping and vibration pathways in a combined steam valve, synthesized from Refs. [31,32,34,35,36,37,40,41].

Taken together, Section 5.1, Section 5.2, Section 5.3 and Section 5.4 define a closed causal sequence. Operating history sets the thermal and flow boundaries; material state and constraint convert those boundaries into displacement; displacement changes clearance and contact; and contact determines actuation margin. This sequence also identifies where materials selection, design, monitoring, and operation can intervene (Figure 6).

## 6. Modeling, Validation, and Uncertainty

### 6.1. A Hierarchy of Thermal and Flow Models

No single resolution is optimal for every decision. One-dimensional Green-function or transfer function models are fast enough for online thermal stress screening but cannot resolve local contact. Two-dimensional axisymmetric finite element (FE) models efficiently represent symmetric sleeves and guides. Three-dimensional FE models are required when ports, impingement, supports, wear, or bolting break axisymmetry. CFD or conjugate heat transfer is most valuable for calibrating non-uniform heat transfer coefficients and wall temperature histories at critical surfaces, rather than for simulating every service cycle.

### 6.2. Constitutive and Contact Modeling

Thermo-elastic analysis is useful for screening, but flexible service may require temperature-dependent plasticity, cyclic hardening or softening, creep, and contact. Continuum mechanics provides the framework for internal variables, creep laws, and damage descriptions [42]. Long-term extrapolation often uses time-temperature parameters such as the Larson–Miller parameter. Thermal fatigue assessment commonly applies strain life concepts derived from Coffin, while rainflow counting and Miner-type summation organize cycle damage [43,44,45,46]. Each method is useful only when its assumptions and interaction rules are stated and calibrated.

Model selection should follow the decision being made (Table 4). For dispatch advice or alarm screening, a calibrated one-dimensional or reduced-order model is preferred because it can update quickly and quantify margins. For design verification of a symmetric guide or sleeve, a two-dimensional thermo-mechanical FE model may be sufficient. For valve bodies with asymmetric ports, non-uniform steam impingement, local repairs, bolted constraints, or suspected jamming, a three-dimensional FE/contact model with sensitivity analysis is required. CFD or conjugate heat transfer should be used when wall heat flux, pressure fluctuation, or lift-dependent jet impingement dominates the boundary condition.

Contact models require as-assembled clearance, interference or preload, thermal contact conductance, friction, and sufficient mesh resolution near contact edges. Friction should be treated as uncertain and state-dependent when oxidation or wear evolves. Creep testing should follow traceable procedures such as ISO 204. Material allowables and pressure boundary checks should use the project-specific edition of the ASME Boiler and Pressure Vessel Code [47,48,49].

### 6.3. Validation, Transferability, and Uncertainty

Validation should target the output used for the engineering decision. Temperature fields can be checked against thermocouples and accessible surface measurements. Deformation and contact require dimensional inspection, stem position, strain, actuator force, or opening and closing time. Damage models require metallography, replicas, hardness, ultrasonics, or other non-destructive evaluation (NDE). Agreement with one casing temperature does not validate an internal clearance prediction.

**Figure 7:**
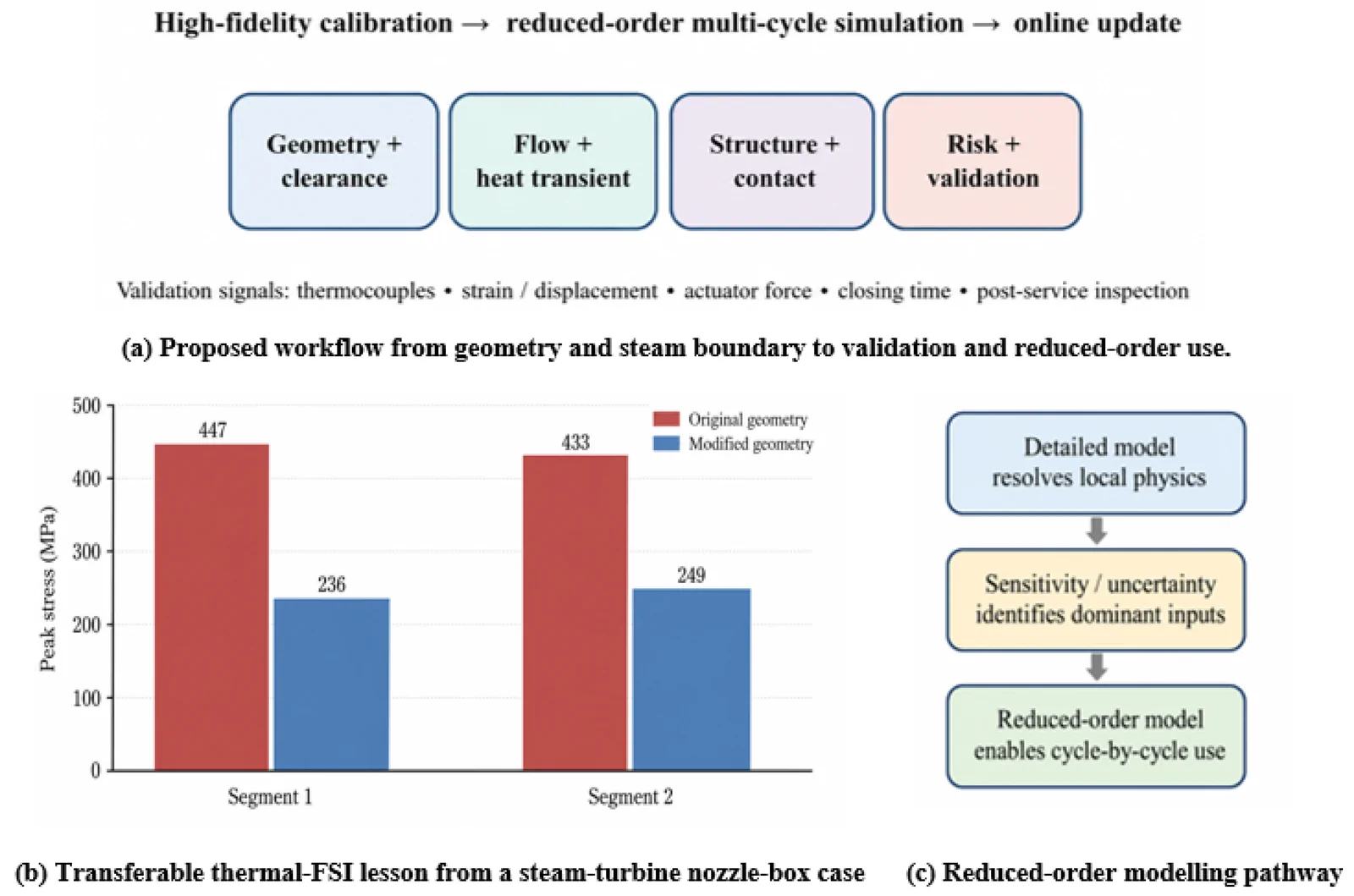
Hierarchical multi-physics assessment using computational fluid dynamics (CFD) and finite element (FE) models [50].

Thermal fluid–structure studies of other turbine hot components show how local geometry and flow modifications can alter both stress and aerodynamic performance. Bryk et al. reported peak-stress reductions from 446.75 to 236.05 MPa and from 432.66 to 249.43 MPa in two modified nozzle-box segments [50]. These results are not valve validation. They instead illustrate a transferable workflow: calibrate flow and thermal fields, assess structure, modify the geometry, and quantitatively re-evaluate the coupled response (Figure 7).

Uncertainty is part of the operating margin, not an optional final allowance. The heat transfer coefficient, initial clearance, friction, material scatter, residual stress, contact stiffness, and shutdown history can each shift the predicted minimum clearance. Sensitivity analysis should first identify dominant variables. Probabilistic or interval methods should then express confidence in the remaining margin. Nominal deterministic contours are useful for mechanism discovery, but they cannot support a plant limit without uncertainty treatment. Model fidelity should therefore be selected according to the decision, validated output, and dominant uncertainty rather than geometric detail alone.

For clearance prediction, uncertainty should be separated into parameter uncertainty, model-form uncertainty, and measurement uncertainty. Parameter uncertainty includes friction coefficient, heat transfer coefficient, creep coefficients, thermal expansion, initial clearance, and residual stress. Model-form uncertainty arises from simplified contact, oxidation, wear, and reduced-order representations. Measurement uncertainty arises from thermocouple placement, actuator force calibration, position sensor resolution, and dimensional inspection repeatability. Reporting these sources separately makes it possible to identify whether additional testing, better instrumentation, or a higher fidelity model would most effectively reduce risk.

## 7. Monitoring, Inspection, and Physics-Informed Digital Twins

### 7.1. Observable Signals and Hidden Valve State

The critical internal state of a hot valve is rarely measured directly. Available signals include steam temperature and pressure, casing temperature, valve position, actuator force or hydraulic pressure, opening and closing time, vibration, and leakage indicators. Online thermal-stress methods combine measured boundary histories with analytical or reduced-order models (ROMs) to infer inaccessible temperatures and stresses [51,52]. Low-cycle fatigue (LCF) monitoring extends this inference to cycle counting and damage accumulation [53].

### 7.2. Inspection as Model Evidence

Inspection supplies evidence unavailable from online monitoring. Dimensional measurements reveal permanent distortion or guide wear; replica metallography and hardness mapping indicate creep or thermal aging; and ultrasonic, magnetic particle, dye-penetrant, or radiographic methods detect different defect classes. Inspection becomes most valuable when its observations update a model state. Measured clearance loss should update geometry, while repair or coating replacement should update material pedigree, surface condition, and residual stress assumptions (Table 5).

### 7.3. From Life Monitors to Valve Digital Twins

Advanced lifetime monitoring systems combine online acquisition, damage calculation, offline analysis, residual life assessment, and model updating [54]. Operation-adaptive creep–fatigue models demonstrate how actual histories can change damage estimates relative to fixed representative cycles [55]. Reviews of turbomachinery digital twins and thermal plant predictive maintenance identify the same enabling elements: validated physics, sensors, data infrastructure, uncertainty treatment, maintenance records, and explicit decision rules [56,57].

**Figure 8:**
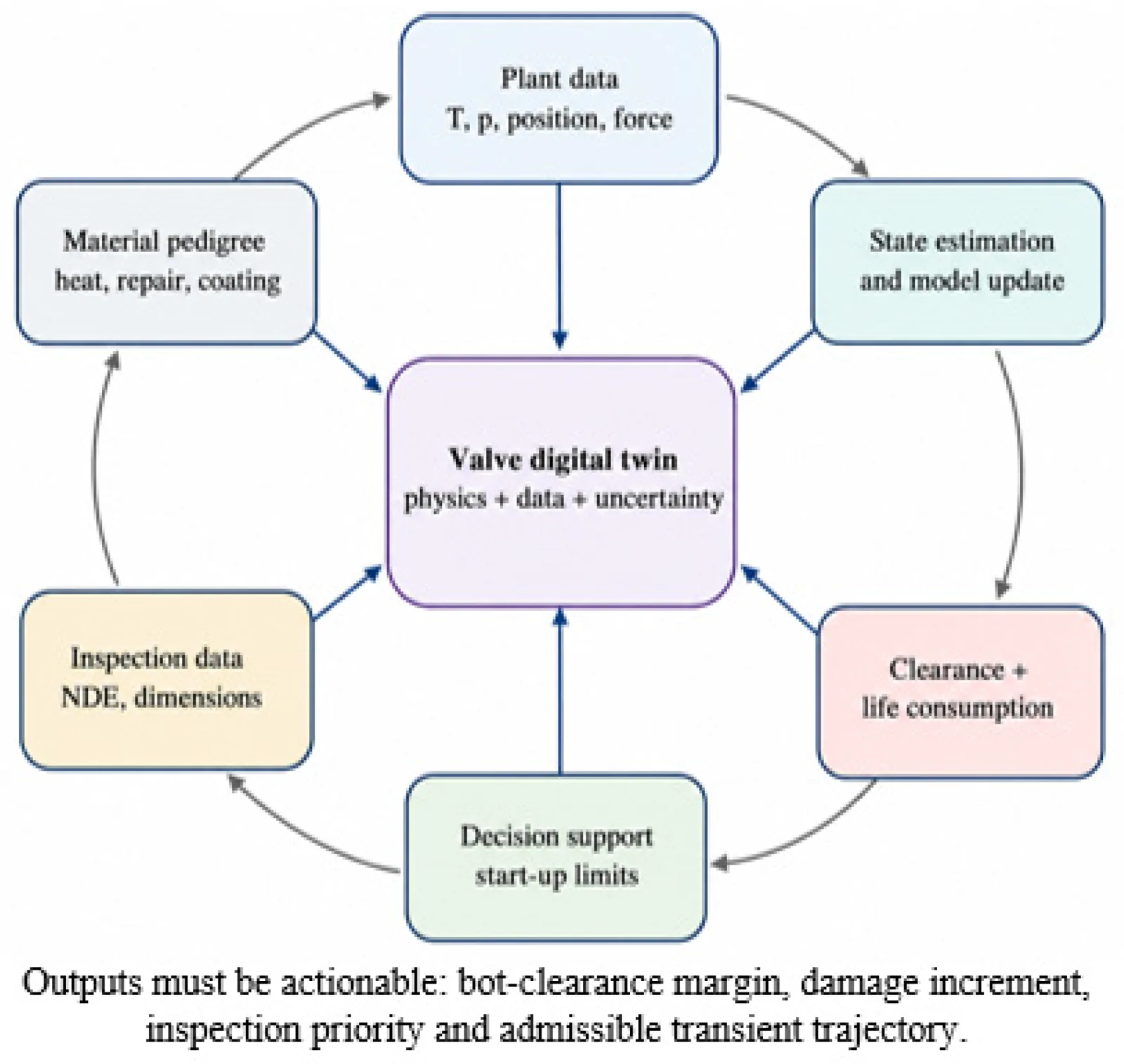
Physics-informed digital twin architecture for main steam valve life management [52,54,56].

For a main steam valve, a useful digital twin is narrower and more actionable than a generic plant dashboard. It should estimate minimum hot clearance margin, residual deformation, creep–fatigue increment, friction or actuation margin, and inspection priority. It should also retain material heat or lot, weld and repair history, and surface treatment state. Figure 8 shows a closed loop in which plant and inspection data update the physics-informed model, while operating decisions define the next transient. This loop converts the causal chain developed above into an auditable life management process.

The minimum experimental information for a reliable valve digital twin should include: measured steam temperature and pressure histories; accessible metal temperatures near thermally critical locations; valve lift, stem position, actuator force or hydraulic pressure histories; room temperature and post-transient clearance measurements; material heat, weld, PWHT, coating, and repair records; and inspection evidence from dimensional checks, NDE, hardness, replicas, or metallography. Validation should proceed from single-transient temperature matching to displacement and actuator force validation, and finally to multi-cycle residual deformation and inspection state updating. Model updating should change only identifiable parameters and should retain uncertainty bounds when different parameter combinations can reproduce the same measured signal [58].

## 8. Co-Design of Materials, Clearance, and Operation

### 8.1. Operation-Side Control

Start-up trajectories should be component-specific because rotor or casing limits do not necessarily protect a valve with smaller moving clearances and more localized steam impingement. Cold starts require control of steam–metal mismatch and strain rate. Hot starts require avoidance of reverse shock and explicit accounting for residual deformation. The preferred trajectory is the fastest one that satisfies both structural damage and functional clearance margins under uncertainty.

### 8.2. Materials and Surface Engineering

For body materials, greater creep strength is beneficial only when toughness, fabrication, and weld performance remain adequate. For stems and guides, dimensional stability and friction may dominate. For seats and faces, the relevant unit of evaluation is the coating substrate–counterface system under steam and thermal cycling. Screening should therefore record debris generation, residual distortion, and post-cycle friction in addition to hardness and mass loss.

### 8.3. Geometry, Clearance, and Assembly

Room temperature nominal clearance is an incomplete design variable. Design should address the distribution of minimum hot clearance across cold, warm, and hot histories, including manufacturing tolerance, wear, oxide growth, and residual deformation. Abrupt stiffness changes and contact edges should be reduced where practical. Interference fits, bolted joints, and supports should be checked for over-constraint. Compliant features may be useful when they promote coordinated expansion without compromising sealing or vibration performance.

Manufacturing and repair scatter should be treated as initial conditions rather than minor workmanship details. Casting or forging distortion, machining tolerance, bushing roundness, stem straightness, coaxiality error, weld shrinkage, hard-facing thickness variation, grinding allowance, and residual stress can shift the contact line before service begins. A robust design should therefore analyze worst-plausible tolerance stacks and repair geometries together with thermal deformation, rather than applying a single nominal clearance to all valves of the same type.

The design objective is predictable deformation, not zero deformation. Optimization should therefore be a coupling of alloy, surface, geometry, clearance, heat transfer, and operating trajectory instead of maximizing one material property. Acceptance testing should likewise include function-oriented state variables and actuation under representative transients, rather than static pressure and room temperature dimensions alone. This co-design view closes the loop between material selection, structural response, and operating control.

## 9. Discussion

The central implication of this short review is that USC steam valve reliability cannot be assessed by a single discipline. Materials degradation controls creep strain, cyclic softening, weld-HAZ weakness, oxidation, and wear; structural constraint converts these material changes into relative displacement; and contact/friction convert displacement into delayed motion or jamming. A credible assessment must therefore preserve the chain from the steam boundary condition to material state, deformation, clearance, contact, and actuation response.

Functional clearance provides the most direct bridge between structural analysis and operability. Its value is not a fixed drawing dimension; it is a time-dependent margin affected by thermal expansion, residual deformation, manufacturing tolerance, oxide scale, wear, debris, repair geometry, and measurement uncertainty. This explains why structural life and functional life can diverge: a valve may remain far from creep rupture while losing enough clearance to show abnormal actuator force or closure delay.

The reviewed modeling hierarchy should be used selectively. High-fidelity CFD-FE contact simulation is justified for design verification, failure reconstruction, or dominant asymmetric heat transfer. Reduced-order and Green function models are more suitable for online monitoring and repeated start-up decisions, but only after calibration against representative thermal, displacement, and actuation signals. The best model is therefore the lowest-fidelity model that predicts the decision variable within a quantified uncertainty band.

**Figure 9:**
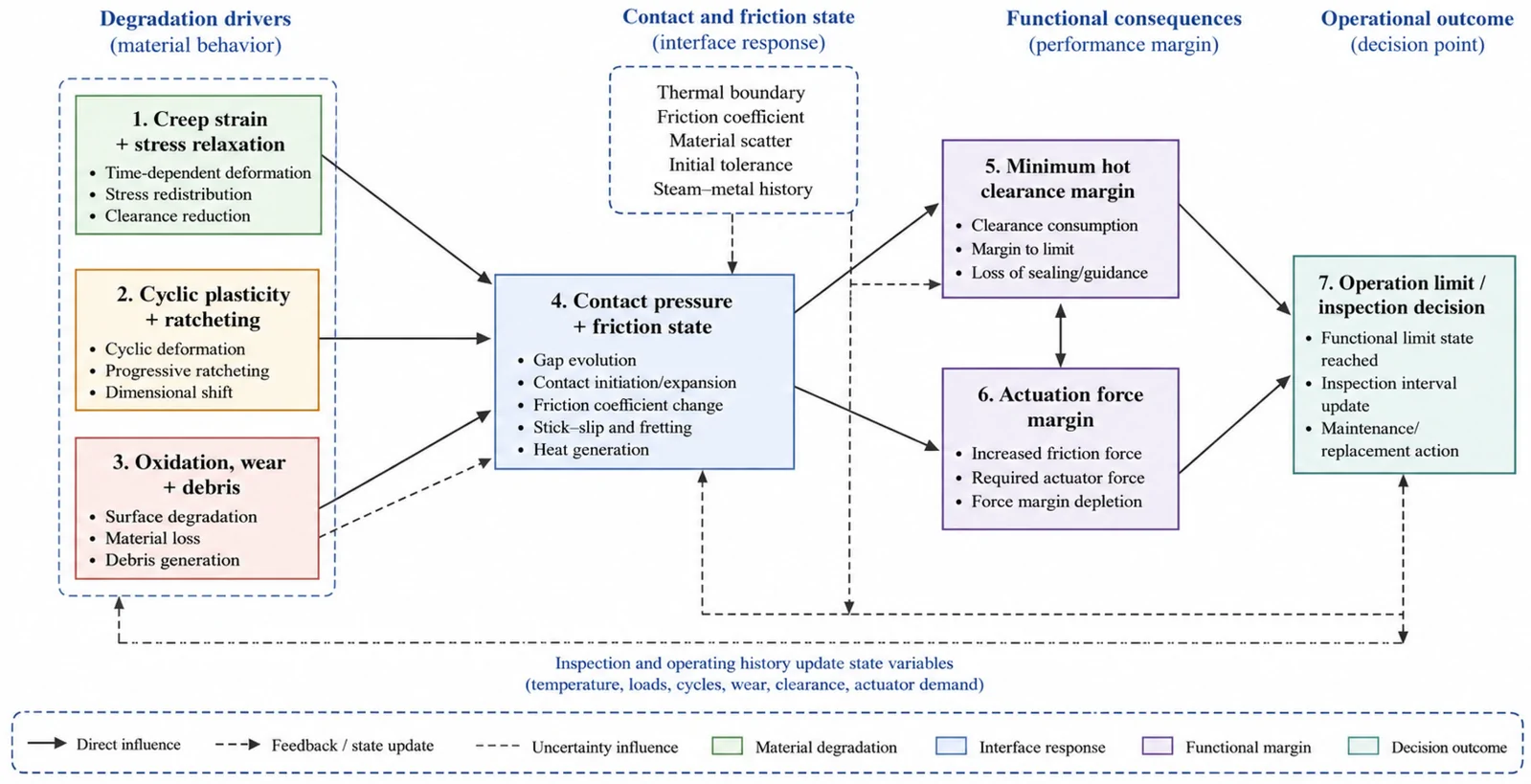
Coupled interaction among creep, cyclic plasticity, oxidation/wear, contact/friction, clearance margin, and actuator demand. Original schematic added in revision.

Advanced material characterization should be tied to model calibration rather than reported as isolated microstructural evidence. EBSD, TEM, atom probe tomography, hardness mapping, creep interrupted tests, replica metallography, and weld-HAZ profiling can identify softening, precipitate coarsening, cavitation, and local property gradients. Cross-domain studies on history-dependent softening and physics-informed constitutive calibration in soft materials and shape-memory polymers (SMPs) are not directly transferable to martensitic steels, but they highlight a useful principle: cyclic, multiaxial, temperature-dependent data and thermodynamic constraints improve the reliability of model updating (Figure 9) [59,60].

**Figure 10:**
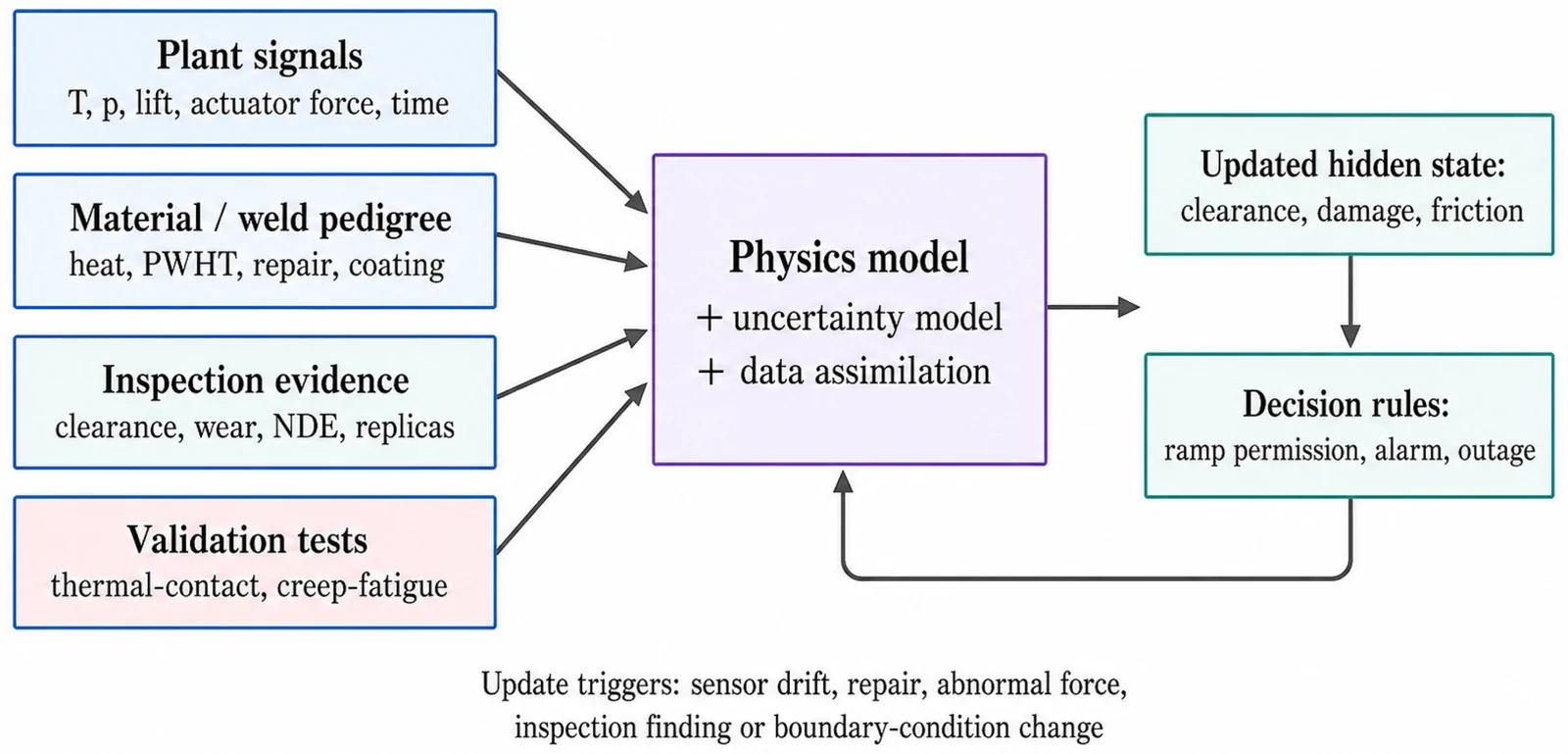
Minimum evidence set and model-updating loop required for a physics-informed digital twin of a main steam valve. Original schematic added in revision.

Digital twins should be interpreted as auditable decision-support systems rather than as generic dashboards. The minimum credible twin must state the measured inputs, hidden state variables, calibration tests, validation targets, uncertainty model, and update rules. Without these items, a digital twin may only reproduce observed temperatures or trends while remaining unvalidated for the quantities that govern jamming risk, namely clearance and actuation margin (Figure 10).

Finally, future work should be prioritized by expected decision impact and technological readiness. Near-term work should focus on valve-level validation datasets, practical clearance/actuator force criteria, and uncertainty-aware reduced models. Medium-term work should develop steam thermal contact tests and repair/weld qualification protocols. Long-term work should integrate validated digital twins with operating optimization and maintenance planning across fleets.

## 10. Research Gaps and Future Directions

The first gap is valve-level validation. Public studies often present contours without complete boundary conditions, material curves, assembly tolerances, or measured actuation behavior. Instrumented experiments and anonymized plant datasets should link steam history with internal or accessible temperature, displacement, actuator force, and post-cycle dimensions. A benchmark geometry with shared material and contact data would permit reproducible comparison among modeling strategies.

The second gap is function-oriented materials testing. Standard creep and fatigue tests remain essential, but they do not reproduce an oxidizing, thermally graded contact with evolving clearance. Thermal contact rigs should combine representative material couples, steam, contact pressure, small-amplitude sliding, dwell, and repeated ramps. Residual displacement, friction evolution, wear debris, and microstructural state should be measured together to calibrate damage and jamming models.

The third gap is uncertainty-aware life management. Initial clearance, the heat transfer coefficient, and friction are often treated as exact, although they can dominate the predicted margin. Future digital twins should propagate uncertainty, track parameter identifiability, and separate model discrepancy from sensor noise. Operating recommendations should be reported as confidence-bounded margins rather than a single deterministic life estimate.

The fourth gap is organizational and analytical integration. Alloy development, hard-facing selection, clearance design, start-up scheduling, and maintenance are commonly optimized by separate groups. A co-design workflow should evaluate their interactions while preserving the distinction between structural and functional life. Table 6 prioritizes the evidence needed to make this integration credible. Together, these priorities define a research program that moves from mechanism identification to validated decision support.

The priorities in Table 6 are ranked by a combination of expected impact on jamming risk decisions, implementation difficulty, and technological readiness. Valve-level validation and functional life criteria are treated as near-term priorities because they directly determine whether simulations and inspections can support operating limits. Thermal contact material tests and uncertainty-aware reduced models are medium-term priorities because they require dedicated rigs and data infrastructure. Integrated materials–clearance–operation co-design has the highest system-level impact but also the highest implementation complexity.

## 11. Conclusions

This review highlights that the reliability of ultra-supercritical (USC) main steam valves cannot be evaluated solely on the basis of pressure boundary strength or conventional creep life. These components should instead be treated as coupled material structure–function systems, in which sealing integrity, stem guidance, contact conditions, dimensional stability, and emergency actuation are controlled by the interaction among thermal loading, material degradation, structural deformation, and assembly clearances.

Cold, warm, and hot starts generate distinct steam–metal temperature histories and therefore different combinations of thermal shock, cyclic deformation, creep accumulation, and residual displacement. However, the severity of a transient cannot be determined from the start-up classification alone. A more reliable assessment requires valve-specific reconstruction of the steam and metal temperature histories, together with the evaluation of retained deformation, reverse thermal shock sensitivity, minimum operating clearance, and contact state evolution.

Material selection for USC main steam valves should consider not only long-term creep rupture strength, but also cyclic softening, creep–fatigue interaction, oxidation, tribological degradation, and the stability of critical dimensions during long-term service. In particular, adequate rupture strength does not necessarily ensure the preservation of sealing performance, guidance accuracy, or functional clearance. Material qualification should therefore be linked directly to the functional requirements of the valve and to the degradation mechanisms expected under realistic operating conditions.

For numerical assessment, credible predictions depend on realistic thermal boundary conditions, temperature-dependent cyclic and creep constitutive properties, actual assembly clearances, contact and friction behavior, and validation targeted to the output of interest. In addition to equivalent stress, creep damage, and fatigue indicators, future studies should report function-related quantities such as minimum hot clearance, residual displacement, local contact pressure, friction state, sealing response, and actuation resistance. Validation should combine temperature measurements, strain or displacement data, inspection results, and, where possible, full-scale or component-level testing.

Physics-informed digital twins provide a promising framework for integrating plant operating signals, inspection records, material pedigree, degradation models, and uncertainty quantification. Nevertheless, their engineering value will depend on the availability of shared valve-level datasets, validated thermal contact models, traceable material information, and clearly defined function-based limit states. At the present stage, digital predictions should be used to support maintenance planning, inspection prioritization, and engineering decision-making rather than to replace physical inspection or expert judgment. Future research should therefore focus on establishing validated function-oriented assessment criteria and on linking material degradation, structural response, and operational performance within a unified life-management framework.

## Figures and Tables

**Table 1:** Comparative implications of cold, warm, and hot starts for main steam valves.

Start Mode	Initial Thermal State	Main Reliability Concern	Key Control Variable
Cold	Low metal temperature after long shutdown	Large steam–metal mismatch; rapid clearance variation; high low-cycle fatigue demand	Steam temperature and pressure ramp rate; allowable strain/stress
Warm	Intermediate retained metal temperature	Moderate thermal gradient; coordinated expansion is possible if the trajectory is controlled	Metal-temperature measurement; steam–metal temperature matching
Hot	High retained metal temperature with possible residual distortion	Reverse thermal shock, residual deformation and clearance loss; potential early jamming	Minimum superheat; residual clearance estimate; actuator margin

**Table 2:** Materials-function relationships for main steam valve components.

Location	Typical Material Strategy	Primary Functional Requirement	Main Degradation and Assessment Focus
Body/chest	Cr–Mo–V or 9–12%Cr heat-resistant steels	Creep strength, toughness, oxidation resistance and manufacturability	Creep strain, thermal fatigue and weld/casting defects; temperature-dependent creep–fatigue and distortion assessment
Stem	Heat-resistant or martensitic stainless steel; surface treatment	Straightness, strength and dimensional stability	Bending, mismatch, oxidation and wear; displacement relative to guides and actuator force
Sleeves/bushings	Heat-resistant steel, stainless steel or hardened surface	Low-friction guidance and clearance stability	Galling, debris, local creep and residual distortion; thermal contact tests and clearance evolution
Seat/face	Co- or Ni-based hard facing or coatings	Sealing hot hardness and wear resistance	Cracking, dilution mismatch, oxidation and erosion; thermal contact fatigue and compatibility
Bolts/fitted joints	High-temperature bolting alloys	Preload retention and relaxation resistance	Creep relaxation, leakage and constraint redistribution; assembly level preload and contact assessment

**Table 3:** Mechanisms that consume functional margin and their observable consequences.

Mechanism	Driving Condition	Functional-Margin Consumption and Observable Consequence	Mitigation Focus
Thermal shock	Large steam–metal mismatch or reverse hot-start gradient	High transient stress and plastic strain; rapid casing-temperature change, abnormal actuator force or timing	Temperature matching and ramp rate control
Creep/ratcheting	High-temperature dwell with constraint and repeated cycles	Permanent strain and residual distortion; post-shutdown dimensional change or actuation force trend	Stress reduction, material selection and multi-cycle assessment
Cyclic softening/fatigue	Repeated strain reversals	Reduced cyclic strength and crack initiation; cycle-dependent NDE indications or hysteresis change	Creep–fatigue monitoring and optimized trajectories
Oxidation/wear debris	Steam exposure, thermal cycling and sliding contact	Scale growth, spallation, abrasion or galling; debris, roughness, leakage or friction increase	Compatible surfaces, oxidation control and inspection
Contact constraint	Clearance loss, interference, fitted joints or misalignment	Contact pressure redistribution and frictional locking; delayed motion or increased closing time	Clearance co-design and validated contact model

**Table 4:** Modeling hierarchy for valve deformation and jamming assessment.

Modeling Level	Main Inputs	Main Outputs	Recommended Use and Main Limitation
1D analytical/Green-function model	Steam history and accessible metal temperature history	Approximate temperature and stress	Suitable for rapid screening, alarms and online state estimation; cannot resolve local clearance or contact
Two-dimensional axisymmetric finite element (FE) model	Axisymmetric geometry, temperature/load histories and material data	Temperature, stress and radial displacement	Efficient for guide/sleeve sensitivity studies and parametric analysis; unsuitable for asymmetric ports, supports or local constraints
Three-dimensional thermo-mechanical FE model	Full geometry, pressure, contact pairs and structural constraints	Local displacement, stress and contact pressure	Suitable for design verification and failure analysis; computationally expensive and sensitive to input uncertainty
Computational fluid dynamics (CFD)/conjugate heat transfer model	Flow path, lift, turbulence model and steam state	Local wall heat flux and pressure fluctuation	Supports calibration of thermal and dynamic loads; costly for long transient histories
Reduced-order model (ROM)/digital twin	Validated physics models, plant data and inspection data	Online estimates of state, clearance and damage	Supports flexible operation and predictive maintenance; requires governance, validation and periodic updating

**Table 5:** Experimental and inspection techniques for validating transient deformation, contact, actuation, and clearance evolution.

Evidence Target	Candidate Technique	Validation Output	Main Limitation
Transient metal temperature and thermal gradient	Embedded thermocouples, surface thermography, fiber optic temperature sensing where feasible	Boundary condition calibration and thermal model validation	Internal locations may be inaccessible; surface temperatures do not uniquely define internal gradients
Deformation and clearance evolution	Hot/cold dimensional metrology, laser displacement, LVDT/stem position sensing, post-cycle 3D scanning	Residual displacement, minimum clearance proxy, and shakedown or ratcheting trend	Direct hot clearance is difficult; alignment and access strongly affect accuracy
Contact, friction, and actuator demand	Actuator force or hydraulic pressure monitoring, closing time measurement, thermal contact rigs, tribological testing in steam	Contact locking tendency, friction evolution, and functional limit-state calibration	Friction is state-dependent and cannot be transferred without matching surface condition and debris
Material degradation and weld/repair state	Replica metallography, hardness mapping, EBSD/TEM/APT where appropriate, ultrasonic, magnetic particle, dye-penetrant, and radiographic NDE	Creep aging, HAZ softening, cavity initiation, cracking, and repair-induced heterogeneity	Local evidence must be linked to valve-level stress, temperature, and contact history

**Table 6:** Research roadmap for materials-informed valve reliability.

Priority	Current Limitation	Recommended Work	Expected Output and Decision Support
Valve-level validation	Limited public data linking steam history, internal state, and actuation	Conduct instrumented full-scale or representative valve tests; use anonymized plant data	Benchmark datasets and validation metrics for model selection and calibration
Thermal contact material tests	Specimen tests often omit contact, oxidation, and clearance effects	Perform steam thermal-cycling tests with realistic material couples and sliding/contact conditions	Friction, wear, residual displacement and microstructure laws for surface/material qualification
Functional life criteria	Assessment is dominated by strength or damage indicators	Correlate clearance, contact pressure and actuator force with closure performance	Calibrated functional limit states for inspection planning and operating limits
Uncertainty-aware reduced models	Simulations are often nominal and deterministic	Introduce global sensitivity analysis, Bayesian/interval updating and model discrepancy treatment	Confidence-bounded state estimates for risk-based start-up decisions
Materials–clearance–operation co-design	Materials, structure and operation are optimized separately	Conduct multi-objective optimization with fabrication and maintenance constraints	Pareto design operation maps for robust flexible service design

## Data Availability

No new data were created or analyzed in this study. Data sharing is not applicable to this article.

## Abbreviations

USC, ultra-supercritical; CFD, computational fluid dynamics; FE, finite element; FSI, fluid–structure interaction; LCF, low-cycle fatigue; NDE, non-destructive evaluation; ROM, reduced-order model.
